# A NEW NORMAL: EXAMINING THE LINKAGES BETWEEN SOCIAL TECHNOLOGY AND LONELINESS

**DOI:** 10.1093/geroni/igac059.2621

**Published:** 2022-12-20

**Authors:** Yeon Ji Ryou, Natasha Peterson, Mengya Wang, Joseph Svec

**Affiliations:** Iowa State University, Ames, Iowa, United States; Iowa State University, Ames, Iowa, United States; Iowa State University, Ames, Iowa, United States; Iowa state university, Ames, Iowa, United States

## Abstract

As the COVID-19 pandemic continues to shape social landscapes, social isolation and loneliness are major issues with mental and physical ramifications. In recent years, individuals of all ages are turning to social technology (ST) to communicate with loved ones. However, the viability of ST as a substitute to in-person interaction remains hotly debated. Moreover, few studies examined how psychosocial factors interact with ST in mental health outcomes. Using the 2020 survey data (N = 1,969) from Health and Retirement Study (HRS), this research examines whether and to what extent ST ameliorates loneliness among 65+ individuals. We identify which personality dimensions moderate the relationship between ST and loneliness using the conceptual framework of the five-factor and the unified theory of acceptance and use of technology model. Linear regression analyses are conducted to determine direct and interaction effects. Results indicate that greater ST use corresponds negatively with loneliness. We also observe that particular personality traits (e.g. extraversion) are negatively associated with loneliness, while neuroticism corresponds positively with loneliness. In the interaction between neuroticism and ST, neuroticism mitigates the association between social technology and loneliness. These findings indicate that ST can be a positive source of social connectivity but the extent may be conditioned on personality profiles. Plausibly, neurotic individuals may exacerbate behavioral propensities by technology use. As evidenced by the robust independent effects of ST and conditional nature of technology connections with neurotic profiles. These findings imply that future intervention should consider individual differences when developing mental health programs using ST.

